# Empirical comparison of methods for analyzing multiple time-to-event outcomes in a non-inferiority trial: a breast cancer study

**DOI:** 10.1186/1471-2288-13-44

**Published:** 2013-03-21

**Authors:** Sameer Parpia, Lehana Thabane, Jim A Julian, Timothy J Whelan, Mark N Levine

**Affiliations:** 1 Ontario Clinical Oncology Group, Department of Oncology, McMaster University, 711 Concession Street – G (60) Wing 1st Floor, Hamilton, ON, L8V 1C3, Canada; 2 Centre of Evaluation of Medicines, St Joseph’s Healthcare—Hamilton, 50 Charlton Avenue East, Hamilton, ON, L8N 4A6, Canada; 3 Juravinski Cancer Centre, 699 Concession Street, Hamilton, ON, L8V 5C2, Canada; 4 Department of Clinical Epidemiology and Biostatistics, McMaster University, Hamilton, ON, Canada

**Keywords:** Non-inferiority, Cox model, Correlation, Marginal model, Frailty model, Competing risks

## Abstract

**Background:**

Subjects with breast cancer enrolled in trials may experience multiple events such as local recurrence, distant recurrence or death. These events are not independent; the occurrence of one may increase the risk of another, or prevent another from occurring. The most commonly used Cox proportional hazards (Cox-PH) model ignores the relationships between events, resulting in a potential impact on the treatment effect and conclusions. The use of statistical methods to analyze multiple time-to-event events has mainly been focused on superiority trials. However, their application to non-inferiority trials is limited. We evaluate four statistical methods for multiple time-to-event endpoints in the context of a non-inferiority trial.

**Methods:**

Three methods for analyzing multiple events data, namely, i) the competing risks (CR) model, ii) the marginal model, and iii) the frailty model were compared with the Cox-PH model using data from a previously-reported non-inferiority trial comparing hypofractionated radiotherapy with conventional radiotherapy for the prevention of local recurrence in patients with early stage breast cancer who had undergone breast conserving surgery. These methods were also compared using two simulated examples, scenario A where the hazards for distant recurrence and death were higher in the control group, and scenario B. where the hazards of distant recurrence and death were higher in the experimental group. Both scenarios were designed to have a non-inferiority margin of 1.50.

**Results:**

In the breast cancer trial, the methods produced primary outcome results similar to those using the Cox-PH model: namely, a local recurrence hazard ratio (HR) of 0.95 and a 95% confidence interval (CI) of 0.62 to 1.46. In Scenario A, non-inferiority was observed with the Cox-PH model (HR = 1.04; CI of 0.80 to 1.35), but not with the CR model (HR = 1.37; CI of 1.06 to 1.79), and the average marginal and frailty model showed a positive effect of the experimental treatment. The results in Scenario A contrasted with Scenario B with non-inferiority being observed with the CR model (HR = 1.10; CI of 0.87 to 1.39), but not with the Cox-PH model (HR = 1.46; CI of 1.15 to 1.85), and the marginal and frailty model showed a negative effect of the experimental treatment.

**Conclusion:**

When subjects are at risk for multiple events in non-inferiority trials, researchers need to consider using the CR, marginal and frailty models in addition to the Cox-PH model in order to provide additional information in describing the disease process and to assess the robustness of the results. In the presence of competing risks, the Cox-PH model is appropriate for investigating the biologic effect of treatment, whereas the CR models yields the actual effect of treatment in the study.

## Background

Randomized controlled trials are considered to be the gold standard for evaluating therapeutic interventions in many different diseases including those in oncology. Unlike studies in other diseases, cancer trials typically follow subjects beyond the planned intervention, often for many years. During this time, subjects may be at risk for several events. For example, subjects in breast cancer trials can experience local recurrence in the treated breast, distant recurrence, death or a combination of these. In most trials, only one of these events is considered the primary outcome and the others are secondary outcomes. The occurrence of multiple events per subject over a period of time is sometimes referred to as event history data [1].

One of the most commonly used statistical approaches for analyzing such data is the Cox proportional hazards (Cox-PH) model, which models the time from randomization to a specific event [2]. However, analyzing each outcome separately using the Cox-PH model does not make use of all the available information because it fails to account for the plausible relationships or correlations between events. For instance, it is possible that experiencing one event increases the risk of experiencing another event. Conversely, it is also possible that the occurrence of one event may even prevent others from occurring, a situation known as competing risks [3]. Standard survival analysis techniques have been shown to bias results in such circumstances [4-6]. The effect of treatment may differ depending on whether or not intermediary events are incorporated into the analysis.

Several statistical methods exist to analyze event history data. These include: the Cox-PH models [2], the competing risk (CR) model [7], the marginal model [8], and the frailty model [9]. The majority of research has focused on using these methods in the analysis of superiority trials where the intervention is expected to be superior to the standard treatment, but their application to non-inferiority trials is lacking. Marginal and frailty models are efficient methods of estimating treatment effect in studies where patients have multiple events of the same type, such as recurrence of asthma attacks. In addition, they are used in studies where treatment can have an effect on multiple events using the same biological pathway. Research on the CR models in superiority trials has shown the Kaplan-Meier approach over-estimates the event rate in the presence of competing risks. However, the relative treatment effect from the CR model remains unchanged compared to the Cox-PH model unless treatment affects the competing event [10].

Non-inferiority randomized trials generally compare the standard treatment with a new treatment that is expected to be less toxic or less expensive or less invasive but “no worse” within a tolerance margin than the standard treatment in terms of clinical outcome.

The purpose of this manuscript is to compare empirically these different approaches in the analysis of a non-inferiority trial in which a subject can experience more than one type of outcome event. In addition, we compare these methods using simulated examples of trials. We first provide a brief overview of the methods, and then apply them to a previously-reported randomized trial of hypofractionated radiotherapy in patients with breast cancer [11,12], and to the simulated trial examples. For the purposes of this study, we will consider the Cox-PH model for each type of event as the primary analysis.

## Methods

### Cox proportional hazards (PH) model

The instantaneous rate of failure known as the hazard rate is defined as the probability of failing in the next small time interval, given that one has already survived until the beginning of the interval [13]. The standard Cox-PH model has become the most frequently used method for modeling hazards and covariates. The model does not make a distributional assumption about the baseline hazard, and assumes that the covariate acts multiplicatively on the hazard independent of time. The model is given by the following:

$$\lambda_{i}\left(t|X\right)=\lambda_{0}\left(t\right)\text{exp}\left(\mathrm{\beta X}\right)$$

where *λ*_*i*_(*t*|*X*) is the hazard of subject *i* conditional on covariate *X* at time *t*, *λ*_0_(*t*) is the baseline hazard at time *t*, *X* is the covariate (e.g. 1 = experimental group, 0 = control group), and *β* is the coefficient representing the effect of treatment independent of time. In cancer trials, the constant treatment effect is represented by the ratio of hazards for the experimental group relative to the control group, or hazard ratio (HR), given by exp(*β*).

### Competing risks (CR) model

Kalbfleisch and Prentice developed the cumulative incidence function (CIF) to analyze competing risk data [14]. The CIF estimates the hazard of an event of interest in the presence of other competing events, known as hazard of the sub-distribution. The estimation of the CIF is similar to that used by Kaplan-Meier; however, the CIF does not censor subjects who experience a competing event and therefore does not require the assumption of independence between the event of interest *j* and the competing events [4,15]. Fine and Gray [7] proposed a proportional hazards model that models the effects of covariates on the hazards of the CIF by distinguishing between competing events and truly censored subjects [15]. Similar to the Cox-PH model, the CR model is given by:

$$\lambda_{ij}^{\text{sub}}(t|X)=\lambda_{0j}^{\text{sub}}\left(t\right)\text{exp}\left(\beta_{j}^{\text{sub}}X\right)$$

where $\lambda_{ij}^{\text{sub}}\left(t|X\right)$ is the hazard of the sub-distribution for cause *j*; $\lambda_{0j}^{\text{sub}}\left(t\right)$ is the baseline hazard of the sub-distribution; and $\beta_{j}^{\text{sub}}$ is the treatment effect of the sub-distribution. This model reduces to the standard Cox-PH model when competing risks are absent.

### Marginal model

Wei, Lin and Weissfeld [8] proposed a marginal model (WLW model) where a subject is assumed to be simultaneously at risk for all events, and is at risk for each event until this event occurs [16]. The WLW model estimates the treatment effect using independent Cox-PH models for each event, and, therefore, the relationship structure between event times does not need to be known [17,18]. For each event *j* for subject *i*, the model is given by

$$\lambda_{ij}\left(t|X\right)=\lambda_{0j}\left(t\right)\text{exp}\left(\beta_{j}X\right)$$

Stratification by event *j* allows for varying underlying baseline hazards *λ*_0*j*_ for each event. In addition, treatment by event interactions allows for estimation of event-specific treatment effects [18,19]. The WLW model also estimates the ‘average effect’ of treatment $\bar{\beta }$ on all events using a weighted average of ${\hat{\beta }}_{j}$, which we will call the average WLW model. Dependencies between observed event times are adjusted for by the use of a robust sandwich estimate of the variance. In the presence of competing risks, the WLW model models both the marginal hazard for death and the cause-specific hazard for recurrences [19].

### Frailty model

Frailty models are survival random effects models in which a parameter for heterogeneity is incorporated into the model. The model is given by:

$$\lambda_{i}\left(t|X\right)=\lambda_{0}\left(t\right)\text{exp}\left(\mathrm{\beta X}+\gamma_{i}\right)$$

where *γ*_*i*_ is the frailty parameter that can also be used to model associations between event times [20]. A large parameter value corresponds to a large correlation between event times for a subject, and also describes the *frailty* or excess risk within a subject [9,21,22]. This model assumes that event times within a subject are independent given the frailty parameter [20]. Similar to other random effects model, this one also yields effects specific to the subjects in the trial. Several published books provide excellent reviews on frailty models [9,21,23].

### The hypofractionation trial

Between April 1993 and September 1996, 1234 patients with early stage breast cancer who had undergone breast conserving surgery were randomly allocated to receive either 42.5 Gray of radiotherapy in 16 fractions (the experimental arm) or 50 Gray in 25 fractions (the standard arm) to the breast for the prevention of local breast recurrence; details and long-term results are described elsewhere [11,12]. The primary outcome of local recurrence was compared was using a point-in-time comparison of local recurrence failure probabilities at five and 10 years [11,12].

For the purpose of this paper, HRs rather than point-in-time failure probabilities will be used. The hypofractionation trial was designed with a control arm local recurrence rate of 7% at 5 years. The non-inferiority margin was set at 5% to tolerate an increase in local recurrence to 12% in the experimental arm. This translates into a HR = *ln*(0.88)/*ln*(0.93) = 1.76. Additional events of interest were distant recurrence, new primary cancer and death. Because of the difficulty in differentiating new primaries from distant recurrences, these will be combined in the distant recurrence category. In addition, we consider only the first occurrence of each type of event.

### Simulated examples

Suppose that a randomized non-inferiority trial similar to the Hypofractionation Trial were designed to demonstrate that an experimental therapy *E* is as good as a control therapy *C* for the prevention of local recurrence in a subset of breast cancer patients. Assuming that the rate of local recurrence at five years in the control arm is 10.0%, and that the maximum tolerable rate of local recurrence at five years in the experimental arm is 14.6% (HR = 1.50), then 1000 patients per treatment arm would be required, giving 90% power and a one-sided alpha of 0.025.

As with the Hypofractionation trial, we assume that these patients will also be at risk for distant recurrence and death. We simulated two possible outcome scenarios (A and B) for this trial using a latent failure time approach. For each treatment group, data were generated using two independent bivariate exponential models based on the hazards in Table 1[24]; one model for local recurrence (*l*) and death (*d*_1_) with correlation of 0.2, and the other for distant recurrence (*m*) and death (*d*_2_) with correlation of 0.6. Time of death (*d*) is given by:

$$d=\left\{\begin{array}{c} d_{1}\\ d_{2}\\ d_{1}\\ d_{2} \end{array}\right)\;\begin{array}{c} \mathrm{if}\min\left(l,d_{1},m,d_{2}\right)=d_{1}\\ \mathrm{if}\min\left(l,d_{1},m,d_{2}\right)=d_{2}\\ \mathrm{if}\min\left(l,d_{1},m,d_{2}\right)=l\\ \mathrm{if}\min\left(l,d_{1},m,d_{2}\right)=m \end{array}$$

which essentially is the time of death if death is the first event, or the time of death that is linked to the first recurrence (local or distant). Independent censoring was generated so that approximately 40% of the observations were censored. Survival times for event and censored observations were calculated for each subject by combining event times and censoring times. Recurrences could occur only prior do death (i.e. recurrence times were less than the death time). Similarly, local recurrence could not occur after distant recurrence. Censoring could occur prior to any events occurring, or after recurrences have occurred. Based on the standard error estimated from the Cox-PH model using hypofractionation trial data, 1000 simulations would produce an estimate to within at least 1.5 percent of the true coefficient.

**Table 1:** Hazards for simulated scenarios of non-inferiority trials (LR = local recurrence, MR = distant recurrence, DT = Death)

**Scenario**	**Outcome**	**Hazard Rate**		**Marginal**
		**Experimental**	**Control**	**Hazard**
				**Ratio**
	LR	0.02	0.02	1.00
A	MR	0.02	0.03	0.67
	DT	0.02	0.04	0.50
	LR	0.03	0.02	1.50
B	MR	0.03	0.02	1.50
	DT	0.04	0.02	2.00

### Analysis

For the Cox-PH model, we structured the data in a “wide” format (i.e. one record per subject). We fit Cox-PH models for each event separately. For the local recurrence model, death and distant recurrences are censored, and for the distant recurrence model, death is treated as a censored observation and local recurrence is ignored. Any recurrence is ignored for the death model. Similarly, for the CR approach, we fit Fine and Gray’s model [7] for each event. Death and distant recurrences are treated as competing events for the local recurrence model, and death is treated similarly for the distant recurrence model. The analysis for death is equivalent to the standard Cox-PH model because death is always observable.

Data for the WLW model is set up in a “long format” where every subject has three records, one for each event, whether censored or observed. The events are treated as independent strata in the model, and time is expressed from randomization to each event. Table 2 shows the time and censoring mechanism for each event given a subject’s event experience.

**Table 2:** Data structure for the WLW model for all possible combinations of events (L = time to local recurrence, M = time to distant recurrence, D = time to death, E = time at end of follow-up, + = censoring indicator)

	**Event Stratum**		
**Events**	**Local**	**Distant**	**Death**
L, M, D	L	M	D
L, M	L	M	E^+^
L, D	L	D^+^	D
M, D	M^+^	M	D
L	L	E^+^	E^+^
M	M^+^	M	E^+^
D	D^+^	D^+^	D

The frailty model is fit using an extension of the Cox-PH model that includes the frailty parameter that assumes a gamma distribution because the events are assumed to be positively correlated [25,26]. For this analysis, every subject has at least one record representing vital status (i.e. alive or dead) at the end of the study, and each recurrence is represented by an additional record.

For the simulation, the HRs and the standard errors of the HRs were averaged on the log scale (1000 replications). All analyses were performed using SAS 9.2 (SAS Institute, Cary, NC) and R 2.13 (http://www.r-project.org).

## Results

### The hypofractionation trial

**Figure 1:**
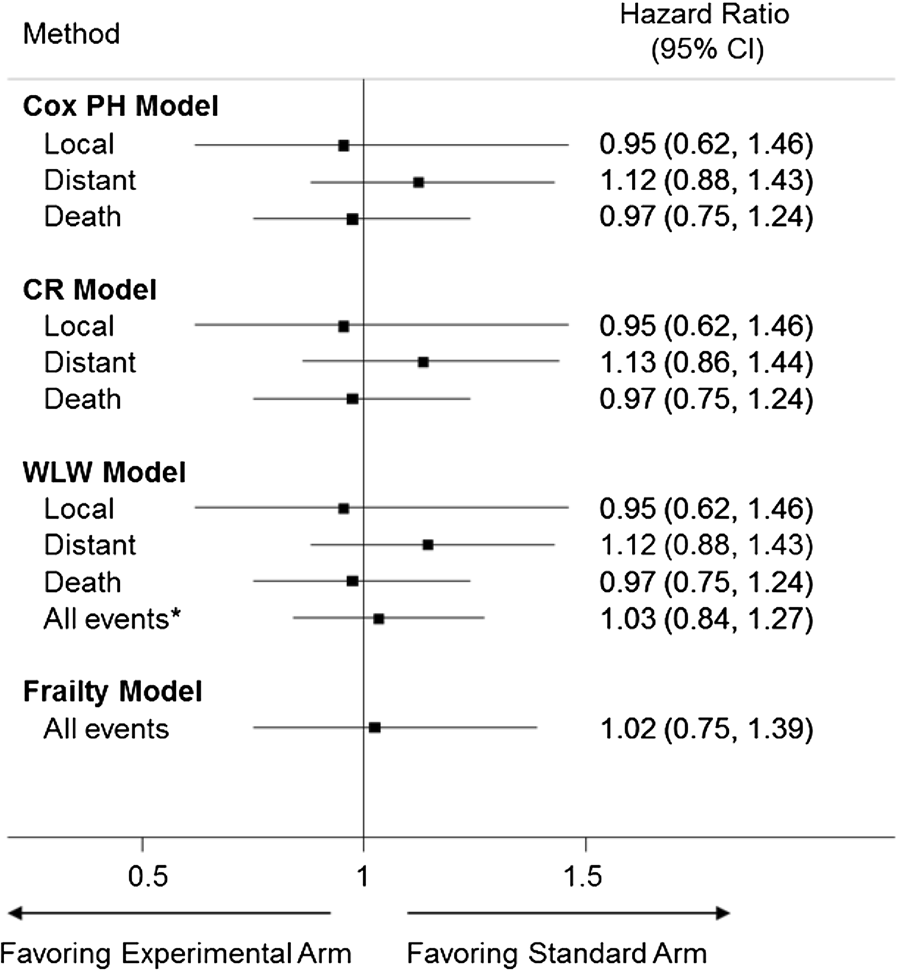
**Forest plot showing the treatment effect in the Hypofractionation Trial using each of the analysis methods (PH = proportional hazards, CR = competing risks, WLW = Wei, Lin and Weissfeld, CI = confidence interval).** * average WLW model.

Figure 1 shows the results of the treatment effect using each of the methods. The Cox-PH, CR and WLW models all yield almost identical estimates for each of the events of interest. The shorter experimental treatment does not affect the occurrence of local recurrence, distant recurrence or death with HRs (95% CI) of 0.95 (0.62, 1.46), 1.12 (0.86, 1.43) and 0.97 (0.75, 1.24) respectively. Since the upper 95% CI of the HR for local recurrence is less than 1.76, non-inferiority can be concluded. Moreover, the frailty and WLW models also show that treatment does not affect the risk of failure from all events combined.

### Simulated examples

**Figure 2:**
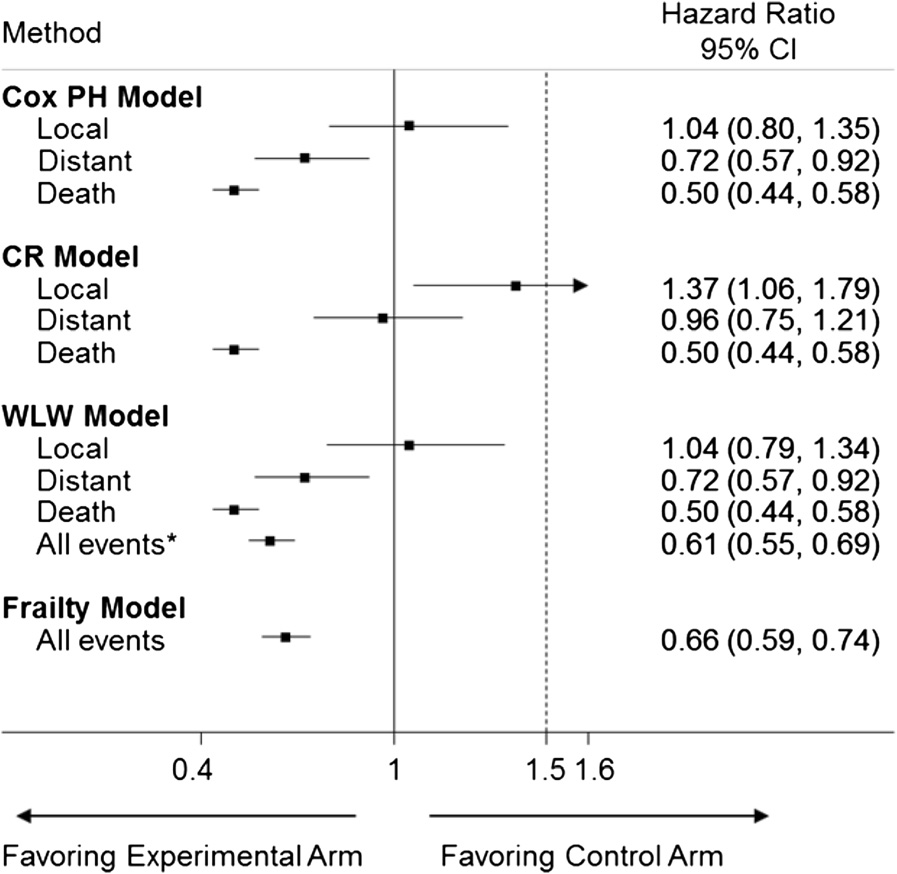
**Forest plot showing the treatment effect in Scenario A using each of the analysis methods (PH = proportional hazards, CR = competing risks, WLW = Wei, Lin and Weissfeld, CI = confidence interval).** * average WLW model.

Results of scenario A (Figure 2), the Cox-PH and WLW model yield an upper 95% CI of 1.35 for the HR for local recurrence, thus suggesting that the experimental therapy is non-inferior to the control. These models also suggest a protective effect of experimental therapy on distant recurrence and death. In contrast, the CR model shows that the experimental therapy is not non-inferior to the control with the upper confidence limit of 1.79 crossing the 1.50 margin. In addition, the CR model yields a treatment effect of 0.96 (0.75, 1.21) for distant recurrence. The frailty and average WLW model show that the experimental arm is significantly protective for all events combined.

**Figure 3:**
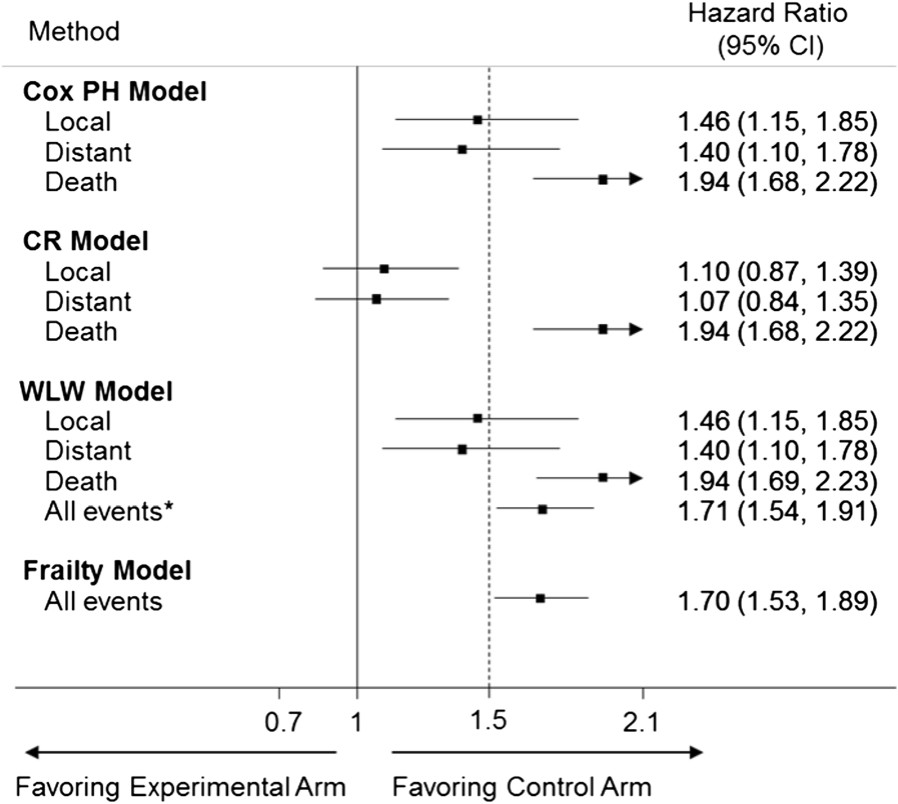
**Forest plot showing the treatment effect in Scenario B using each of the analysis methods (PH = proportional hazards, CR = competing risks, WLW = Wei, Lin and Weissfeld, CI = confidence interval).** * average WLW model.

The results of Scenario B (Figure 3) are opposite to that of scenario A. In this case, the Cox-PH and WLW models yield an upper 95% CI for the HR for local recurrence that is greater than the 1.50 margin. On the other hand, the CR model shows that the experimental therapy is non-inferior to the control with respect to local recurrence. Moreover, the CR model shows that treatment has no effect on distant recurrence whereas the Cox-PH and WLW models suggest a detrimental effect of the experimental treatment on distant recurrence. The frailty and average WLW model suggest that the experimental treatment is harmful when considering all events together.

## Discussion

In non-inferiority clinical trials of patients with breast cancer, patients may be at risk of and may experience multiple failure types. The occurrence of one of these events may alter the probability of occurrence of other events. Moreover, the influence of treatment may differ depending on whether another event has occurred, thus affecting the conclusions of the trial. This paper discusses, and applies four approaches of analyzing non-inferiority trials with multiple events, by using data from an existing trial in which subjects with breast cancer could experience local recurrence, distant recurrence, death, or a combination of these events. In addition, we compared the methods using simulated examples of non-inferiority trials.

The analysis of the Hypofractionation Trial showed that treatment was not associated with increased risk of any of the events of interest either individually or in combination. The results for each event using the Cox-PH model and the CR model are similar, suggesting that the impact of competing risks in this data set is minimal. The treatment estimates for each event from the WLW model are identical to those of the standard Cox-PH model since the estimates of the regression coefficients are calculated using equivalent methods. However, the adjustment of correlation in the variance estimate of the WLW model leads to slightly different confidence intervals when compared with the Cox-PH model. The WLW model is also susceptible to the competing risk problem since subjects are at risk for events until they occur, but the model yields unbiased estimates when treatment does not influence the competing events [27].

Scenarios A and B provide evidence that the presence of multiple events could alter the conclusions of the trial depending on the method of final analysis. The Cox-PH and WLW local recurrence models ignore the hazards for distant recurrence and death, thus resulting in different conclusions for local recurrence when compared to with CR model. Similarly, the Cox-PH and WLW distant recurrence models ignore the hazard for death. By ignoring the competing risks, the Cox-PH and WLW methods model the cause-specific hazard or the marginal failure times, and the effect of treatment can be interpreted as the “pure effect” or the biologic effect of treatment on the event of interest [28]. This is the effect of treatment under the assumption that the competing risk had not occurred, which can be of interest to investigators.

Unlike the Cox-PH and WLW models, the CR model does not censor patients who have had a distant recurrence or death, but rather assumes that these patients will have a zero risk of local recurrence once distant recurrence or death is observed. Censoring assumes that the patient is still at risk for local recurrence. Therefore, in the CR model, the treatment group with higher relative hazards of distant recurrence and death will have a relatively lower hazard of local recurrence, and the HR for local recurrence will favor this treatment group. This approach models the hazard of the sub-distribution, and the effect of treatment can be described as the “real effect” or the actual effect seen in the data [28,29].

The CR model does provide additional information about the treatment when competing events are present. The Cox-PH model declares non-inferiority of local recurrence, but the CR model shows that the absolute effect of treatment is inferior in the study because the control group has a higher hazard of competing events (scenario A). However, in some situations (scenario B),the results from the CR model should be interpreted with caution since the CR model may show that the experimental group is non-inferior to the control for local recurrence, but at the expense of increased distant recurrence or death, which are clinically worse outcomes. If this is a concern, one may opt to design the trial using an outcome such as disease-free survival which encompasses local and distant recurrence. In addition, CR models have less power than the Cox-PH models to rule out the same non-inferiority margin [30].

The average WLW and frailty models are useful in investigating the overall effect of treatment for any event accounting for the correlation between event times in their respective ways. The main advantage of these approaches is that they are efficient in their estimation of regression coefficients due to their ability to use all the data and to adjust for the association between event times, thus increasing statistical power. However, their use is limited when dealing with dissimilar types of events with different clinical etiology such as local and distant recurrence, because the approach does not provide HRs of treatment and other factors in relation to specific events but rather a combination of all events. Moreover, these models do not correspond to the design of the trial which is evaluating a local treatment and based on rate of local recurrence.

The methods behave similarly in non-inferiority trials as compared with superiority trials. As in superiority trials, competing risks is an issue when treatment affects the competing event. When the distribution of competing events are similar in both treatment groups, the CR model and the Cox-PH model yield similar results, and therefore, the biologic effect and actual effect of treatment in the study are similar. However, similar to superiority trials, when treatment has a differential effect on competing events, the results of the biologic and actual effect of treatment can contradict each other.

A limitation of this study is that we compared analytic techniques using a single non-inferiority trial. To overcome this, we simulated examples to illustrate that the choice of method may influence the conclusions. However, we simulated only two scenarios using the latent failure time approach, thus limiting the generalizability of the results. Secondly, we generated the data using a latent failure time approach which is not without controversy [31]. However, we did not use the model or its assumptions in any of our analyses, and do not recommend it for use for analysis. Lastly, we considered only the most commonly used methods of analysis which are readily available in current statistical software. Alternative options include jointly modeling all types of events using a joint frailty model where each event has one hazard function [32], or using a multivariate competing risk frailty model [33]. However, such undertakings would be computationally intensive and complex.

## Conclusions

Our results show that the choice of event-specific models did not affect the non-inferiority conclusion of the Hypofractionation Trial. However, our examples showed that the CR method did yield contrasting conclusions to the Cox-PH and WLW models when competing events were present. In general, the method of analysis should be determined by the research question. The Cox-PH or the WLW model can be used for analysis of non-inferiority trials when the question relates to the biologic effect of treatment. The CR model should also be used when competing risks are present as it provides valuable information on the actual effect of treatment in the study, especially when treatment has an effect on the competing event. Both models should be part of a comprehensive analysis. The frailty and average WLW provide similar results of the overall effect of treatment on all the events. When subjects are at risk for multiple events in non-inferiority trials, researchers should consider the use of the CR, WLW and frailty models concurrent with the standard Cox-PH model in order to provide additional information in describing the disease process.

## Competing interests

The authors declare that they have no competing interests.

## Author’s contributions

SP, JAJ, LT and MNL conceived the study. SP conducted literature review, designed and implemented the simulation, preformed data analysis and wrote the initial draft of the manuscript. TJW, JAJ and MNL participated in the design and implementation of the Hypofractionation Trial. All authors reviewed and revised the draft version of the manuscript. All authors read and approved the final version of the manuscript This research was funded in part by funds from the CANNeCTIN Program.

## Pre-publication history

The pre-publication history for this paper can be accessed here:

http://www.biomedcentral.com/1471-2288/13/44/prepub
